# Association of thyroid hormone with body fat content and lipid metabolism in euthyroid male patients with type 2 diabetes mellitus: a cross-sectional study

**DOI:** 10.1186/s12902-021-00903-6

**Published:** 2021-12-06

**Authors:** Xia Sun, Liping Chen, Rongzhen Wu, Dan Zhang, Yinhui He

**Affiliations:** 1 Department of Endocrinology, Lishui Hospital of Traditional Chinese Medicine, No. 800 Zhongshan Street, Liandu District, Lishui, Zhejiang, 323000 China; 2 Department of Cardiovascular Medicine, Lishui Hospital of Traditional Chinese Medicine, Lishui, Zhejiang, China; 3 Department of Clinical Laboratory, Lishui Municipal Central Hospital, Lishui, Zhejiang, China; 4 Department of Endocrinology, Lishui Municipal Central Hospital, Lishui, Zhejiang, China

**Keywords:** Type 2 diabetes mellitus, Thyroid hormone, Body fat content, Lipid metabolism, Cross-sectional study

## Abstract

**Background:**

This study aimed to explore the associations of thyroid hormones with body fat content and lipid metabolism in euthyroid male patients with type 2 diabetes mellitus (T2DM).

**Methods:**

In January 2017, a cross sectional study, 66 male patients with T2DM who met the World Health Organization diagnostic criteria of 1999 who were ≥ 18.0 years and had normal thyroid function were recruited at a tertiary hospital. The categories of thyroid hormones (free triiodothyronine [FT3], free thyroxine [FT4], and thyroid-stimulating hormone [TSH]) were divided into three groups according to tertiles of thyroid hormones.

**Results:**

The mean FT3, FT4, and TSH of the patients were 2.56 pg/mL, 1.03 ng/dL, and 1.50 μIU/mL, respectively. Increased FT3 were associated with higher body mass index (BMI) (*P* <  0.001), body fat percentage (BFP) (*P* = 0.008), visceral fat content (VFC) (*P* = 0.019), adiponectin (*P* = 0.037), tumor necrosis factor alpha (TNF-α) (*P* <  0.001), and interleukin 6 (IL-6) (*P* = 0.015). There were significant differences among the different FT4 categories for BMI (*P* = 0.033), waist–hip ratio (WHR) (*P* = 0.030), low-density lipoprotein cholesterol (LDL-C) (*P* = 0.014), and IL-6 (*P* = 0.009). Increased TSH could increase the total cholesterol (TC) (*P* = 0.005) and high-density lipoprotein cholesterol (HDL-C) (*P* = 0.010). FT3 was positively correlated with BMI (r = 0.45; *P* <  0.001), WHR (*r* = 0.27; *P* = 0.028), BFP (*r* = 0.33; *P* = 0.007), VFC (*r* = 0.30; *P* = 0.014), adiponectin (r = 0.25; *P* = 0.045), TNF-α (*r* = 0.47; *P* <  0.001), and IL-6 (*r* = 0.32; *P* = 0.008). FT4 was positively correlated with HDL-C (*r* = 0.26; *P* = 0.038), LDL-C (*r* = 0.26; *P* = 0.036), and adiponectin (*r* = 0.28; *P* = 0.023). TSH was positively correlated with TC (*r* = 0.36; *P* = 0.003).

**Conclusion:**

This study found that the changes in thyroid hormones are associated with various body fat content and lipid metabolism in euthyroid male patients with T2DM.

## Background

The prevalence of type 2 diabetes mellitus (T2DM) is a prominent global public health problem, accounting for approximately 415 million cases globally, and the prevalence of T2DM is predicted to increase to 642 million in 2040 [1]. T2DM could induce excessive risks of cardiovascular disease, neuropathy, nephropathy, retinopathy, and microvascular complications. Moreover, the economic burden of diabetes globally was $1.31 trillion and accounted for approximately 1.8% of the global gross domestic product [2]. Studies showed that insulin resistance was common in T2DM and metabolic syndrome [3, 4]. Insulin resistance was a risk factor for stroke and end-stage renal disease [5]. Obesity is the basis of insulin resistance because of the harmful effects of excess fat accumulation on glucose metabolism, which causes functional impairments in metabolic pathways of several areas such as adipose tissue and peripheral organs, like the liver, heart, pancreas, and muscles [6].

Thyroid hormones, including thyroxine and triiodothyronine, regulate the synthesis, mobilization, and breakdown of lipids. Thyroid hormones are closely related to obesity, and slight changes in serum thyroid hormone level can cause local fat accumulation and increased body mass [7, 8]. The thyrotropin receptor (TSHR) has long been considered as a key regulator of thyroid function [9]. Recent studies have shown that thyroid-stimulating hormone (TSH) can also bind directly to TSHR in tissues outside the thyroid to exert external effects, such as in the adipose tissue and liver [10]. The role of thyroid function in energy metabolism has been extensively studied, but traditional viewpoints only emphasize the role of hypothyroidism or subclinical hypothyroidism (SCH) in obesity. Among patients with obesity but with normal thyroid function, thyroid hormones, especially TSH, are significantly different from that of people of healthy weight. This manifests as higher TSH, free triiodothyronine (T3), and free thyroxine (T4) levels in obese patients than those in healthy people [11, 12]. The incidence of thyroid diseases in patients with T2DM is significantly increased, especially SCH, and is more common in women than men [13, 14]. However, the relationship among TSH, FT3, FT4, body fat content, and lipid metabolism in T2DM patients with normal thyroid function has not been reported. Therefore, in this study, we aimed to explore the association of thyroid hormone levels with body fat content and lipid metabolism in euthyroid male patients with T2DM.

## Methods

### Study design and subjects

This cross-sectional study recruited euthyroid male patients with T2DM admitted in a tertiary hospital on January 2017. This study was approved by the ethics committee of Lishui Municipal Central Hospital (ethics no. 2016–26). All patients provided informed consent for the inclusion in the study. Male patients with T2DM who met the World Health Organization diagnostic criteria of 1999 who were ≥ 18.0 years and with normal thyroid function were included. Patients with diabetes with acute complications, pulmonary and cardiac diseases, liver and kidney disorder, or clinical and subclinical thyroid diseases were excluded. Finally, 66 patients were included in the final analysis.

### Data collection

#### Baseline characteristics

Baseline data was collected from all participants, including age, anthropometric, and laboratory measurements. Blood pressure was recorded, with the systolic and diastolic blood pressure measured at sitting position, and mean blood pressure was calculated. After 12 h of fasting overnight, blood from the elbow vein was collected into two sample tubes. The serum was centrifuged from one tube within 2 h, and hematological parameters were determined immediately. Fasting blood glucose (FBG) was detected by hexokinase (Abbott, Abbott Park, IL, USA), fasting insulin (FIns) by chemiluminescence (Abbott), and glycosylated hemoglobin (HbA1C) by ion chromatography (Tosoh, Tokyo, Japan). The insulin resistance index (Homeostatic Model Assessment of Insulin Resistance [HOMA-IR]) was calculated by the minimum homeostasis model. The formula was FBG*FINS/22.5.

#### Thyroid hormones

Blood TSH, FT3, and FT4 were detected by chemiluminescence (Abbott) (normal reference ranges: TSH, 0.34–5.60 μIU/mL; FT3, 1.71–3.71 pg/mL; FT4, 0.70–1.48 ng/dL). The TSH, FT3, and FT4 were divided into 3 groups based on the tertiles of thyroid hormones, and each group included 22 patients.

#### Body fat content

Height (m), weight (kg), waist circumference, and body mass index (BMI; kg/m^2^) were measured by uniformly trained nurses for all subjects. Body fat percentage (BFP) and visceral fat content (VFC) were measured by direct segmental impedance measurement (InBody 720 human body composition analyzer; Baisibeisi Medical Equipment Trading Co., Ltd., Shanghai, China).

#### Lipid metabolism

After 12 h of fasting overnight, total cholesterol (TC) was measured by CHOD-PAP (Zhongya Company, Hangzhou, China), triglyceride (TG) by GPO-PAP (Zhongya Company), and high- and low-density lipoprotein (HDL-C and LDL-C, respectively) by homogeneous direct method (Zhongya Company). After adding anticoagulant to the other tube, the plasma was centrifuged and preserved at − 70 °C for the determination of adiponectin, leptin, visfatin, and tumor necrosis factor alpha (TNF-α). Shanghai Enzyme-linked Biotechnology Co., Ltd. (Shanghai, China) performed the laboratory determination. The standard curves were 0.156–10 ng/mL for plasma adiponectin, 0–50 ng/mL for leptin, 0.1–1000 ng/mL for visfatin, and 2.5–80 pg/mL for plasma TNF-α.

### Statistical analysis

All data were processed by SPSS version 21.0 statistical software (IBM Corp., Armonk, NY, USA). Continuous variables were first tested for normality. Normally distributed continuous variables were expressed by mean ± standard deviation, and multigroup comparison was conducted by analysis of variance and post-test (Student–Newman–Keuls *q* value). Linear correlation analysis was used for the associations of thyroid hormones with body fat content and lipid metabolism in euthyroid male patients with T2DM. All reported *P* values are two-sided, and *P* values < 0.05 were considered statistically significant.

### Patient and public involvement

Patient and public involvement were not involved in the study design.

## Results

### Baseline characteristics

Of 66 included patients, the mean age was 49.70 years. Moreover, the mean FT3, FT4, and TSH levels of the patients were 2.56 ± 0.43 pg/mL, 1.03 ± 0.14 ng/dL, and 1.50 ± 0.74 μIU/mL, respectively. The mean FBG, FIns, HOMA-IR, and HbA1c of patients were 10.07 ± 4.69 mmol/L, 8.32 ± 6.14 uU/mL, 3.21 ± 1.97, and 9.74% ± 2.23%, respectively. The body fat content and lipid metabolism of the patients are summarized in Table 1.

**Table 1:** Baseline characteristics of included patients

Parameter	Baseline
Age	49.70 ± 9.45
TSH	1.50 ± 0.74
FT3	2.56 ± 0.43
FT4	1.03 ± 0.14
FPG	10.07 ± 4.69
Fasting insulin	8.32 ± 6.14
HOMA-IR	3.21 ± 1.97
GGT	48.09 ± 39.61
HbA1c%	9.74 ± 2.23
MAP	97.83 ± 12.81
BMI	25.29 ± 3.40
WHR	0.93 ± 0.05
BFP	23.74 ± 5.82
VFC	110.67 ± 27.28
TG	2.00 ± 1.57
TC	4.72 ± 1.30
HDL	1.03 ± 0.25
LDL	2.72 ± 0.96
Adiponectin	5.35 ± 0.72
Leptin	3.17 ± 0.36
Visfatin	29.14 ± 3.16
TNF-α	17.99 ± 2.52
IL-6	11.59 ± 1.46
Homocysteine	9.30 ± 1.95
UA	325.94 ± 88.21

*BFP body fat percentage, *BMI* body mass index, *FBG* fasting blood glucose, *HDL* high-density lipoprotein, *LDL* low-density lipoprotein, *MAP* mean arterial pressure, *WHR* waist-hip ratio, *TC* total cholesterol, *TG* triglyceride, *UA* uric acid, *VFC* visceral fat content

### FT3

The distribution of the body fat content and lipid metabolism according to FT3 are shown in Table 2. There were significant differences in BMI (*P* <  0.001), BFP (*P* = 0.008), VFC (*P* = 0.019), adiponectin (*P* = 0.037), TNF-α (*P* <  0.001), and IL-6 (*P* = 0.015) among the FT3 categories, but there were no significant differences in waist–hip ratio (WHR) (*P* = 0.125), TG (*P* = 0.184), TC (*P* = 0.383), HDL-C (*P* = 0.082), LDL-C (*P* = 0.336), leptin (*P* = 0.123), visfatin (*P* = 0.566), homocysteine (*P* = 0.951), or uric acid (*P* = 0.869). The pairwise comparison showed the following: the lowest category of FT3 was associated with lower BMI, as compared with the middle (mean difference [MD], − 2.76; 95% confidence interval [CI], − 4.64 to − 0.89; *P* < 0.05) and highest categories of FT3 (MD, − 3.60; 95% CI, − 5.43 to − 1.76; *P* < 0.05); the lowest (MD, − 4.84; 95% CI, − 8.10 to − 1.57; *P* < 0.05) and middle categories of FT3 (MD, − 4.19; 95% CI, − 7.49 to − 0.88; *P* < 0.05) were associated with lower BFP, as compared with the highest category of FT3; the lowest (MD, − 21.51; 95% CI, − 37.02 to − 6.00; *P* < 0.05) and middle categories of FT3 (MD, − 16.39; 95% CI, − 32.09 to − 0.69; *P* < 0.05) were associated with lower VFC, as compared with the highest category of FT3; the lowest category of FT3 was associated with lower adiponectin, as compared with the middle (MD, − 0.52; 95% CI, − 0.95 to − 0.10; *P* < 0.05) and highest categories of FT3 (MD, − 0.43; 95% CI, − 0.84 to − 0.01; *P* < 0.05); the lowest category of FT3 was associated with lower TNF-α, as compared with the middle (MD, − 2.36; 95% CI, − 3.72 to − 1.00; *P* < 0.05) and highest categories of FT3 (MD, − 2.79; 95% CI, − 4.12 to − 1.46; *P* < 0.05); the lowest category of FT3 was associated with lower IL-6, as compared with the middle (MD, − 0.91; 95% CI, − 1.76 to − 0.06; *P* < 0.05) and highest categories of FT3 (MD, − 1.20; 95% CI, − 2.02 to − 0.37; *P* < 0.05). Furthermore, we noted that FT3 was positively correlated with BMI (r = 0.45; *P* < 0.001), WHR (*r* = 0.27; *P* = 0.028), BFP (*r* = 0.33; *P* = 0.007), VFC (*r* = 0.30; *P* = 0.014), adiponectin (*r* = 0.25; *P* = 0.045), TNF-α (*r* = 0.47; *P* < 0.001), and IL-6 (*r* = 0.32; *P* = 0.008) (Table 3).

**Table 2:** The body fat content and lipid metabolism according to free T3, free T4, and TSH

Parameter	Free T3			*P* value	Free T4			*P* value	TSH			*P* value
	(≤2.37)	(2.39–2.74)	(≥2.76)		(≤0.96)	(0.97–1.07)	(≥1.09)		(≤1.09)	(1.11–1.75)	(≥1.88)	
BMI	23.16 (2.38)	25.92 (2.72)^a^	26.75 (3.86)^a^	< 0.001	24.19 (2.93)	26.77 (3.59)^a^	24.92 (3.26)	0.033	24.25 (2.55)	25.42 (4.04)	26.20 (3.32)	0.163
WHR	0.91 (0.05)	0.93 (0.05)	0.95 (0.05)	0.125	0.92 (0.04)	0.95 (0.06)^a^	0.92 (0.05)^b^	0.030	0.91 (0.05)	0.93 (0.05)	0.94 (0.05)	0.163
BFP	21.85 (4.97)	22.50 (5.46)	26.69 (5.95)^a,b^	0.008	21.99 (5.29)	24.83 (5.91)	24.40 (6.10)	0.221	22.66 (5.63)	22.88 (6.04)	25.69 (5.56)	0.158
VFC	101.54 (20.25)	106.66 (26.34)	123.05 (30.29)^a,b^	0.019	104.20 (20.65)	122.16 (29.07)	105.64 (28.70)	0.050	106.66 (23.35)	110.45 (31.19)	114.89 (27.39)	0.613
TG	1.55 (0.87)	2.01 (1.65)	2.41 (1.94)	0.184	2.35 (2.04)	1.71 (1.26)	1.92 (1.31)	0.393	1.91 (1.73)	1.67 (1.12)	2.41 (1.77)	0.291
TC	4.72 (1.47)	4.43 (0.98)	4.98 (1.37)	0.383	4.54 (1.14)	4.38 (1.04)	5.23 (1.56)	0.069	4.04 (0.75)	4.85 (1.28)^a^	5.26 (1.48)^a^	0.005
HDL	0.99 (0.22)	0.97 (0.21)	1.12 (0.30)	0.082	1.00 (0.21)	0.95 (0.24)	1.13 (0.28)	0.068	0.90 (0.19)	1.12 (0.23)^a^	1.06 (0.28)^a^	0.010
LDL	2.93 (1.26)	2.49 (0.65)	2.74 (0.86)	0.336	2.41 (0.75)	2.57 (0.68)	3.20 (1.22)^a,b^	0.014	2.39 (0.74)	2.81 (0.83)	2.98 (1.19)	0.105
Adiponectin	5.03 (0.67)	5.56 (0.71)^a^	5.46 (0.72)^a^	0.037	5.05 (0.62)	5.56 (0.77)	5.43 (0.70)	0.052	5.22 (0.76)	5.33 (0.71)	5.50 (0.71)	0.425
Leptin	3.04 (0.33)	3.25 (0.36)	3.22 (0.38)	0.123	3.07 (0.34)	3.29 (0.35)	3.15 (0.38)	0.129	3.08 (0.40)	3.20 (0.35)	3.23 (0.34)	0.339
Visfatin	29.58 (3.59)	28.56 (3.10)	29.26 (2.82)	0.566	29.98 (3.67)	27.86 (2.73)	29.59 (2.71)	0.058	29.29 (3.45)	29.13 (2.49)	29.01 (3.59)	0.959
TNF-α	16.26 (2.32)	18.63 (2.21)^a^	19.06 (2.16)^a^	< 0.001	17.01 (2.26)	18.76 (2.68)	18.20 (2.40)	0.062	17.24 (2.83)	18.31 (2.32)	18.42 (2.32)	0.234
IL-6	10.88 (1.57)	11.79 (1.47)^a^	12.08 (1.11)^a^	0.015	11.04 (1.57)	12.32 (1.37)^a^	11.40 (1.17)^b^	0.009	11.45 (1.43)	11.39 (1.55)	11.92 (1.41)	0.423
Homocysteine	9.32 (2.52)	9.19 (1.36)	9.37 (1.87)	0.951	9.54 (2.35)	8.72 (1.36)	9.63 (1.97)	0.237	8.87 (1.03)	9.33 (2.01)	9.69 (2.51)	0.381
UA	325.45 (93.30)	333.67 (94.55)	319.35 (80.30)	0.869	335.73 (99.80)	307.73 (89.73)	334.36 (74.49)	0.501	310.41 (98.37)	338.18 (90.82)	329.23 (75.74)	0.574

*a: compared with lowest category of tertile with *P* < 0.05; b: highest versus middle category of tertile with *P* < 0.05

**Table 3:** Correlation analysis of thyroid hormone and body fat content and lipid metabolism

Variable	TSH		Free T3		Free T4	
	*r*	*P* value	r	*P* value	*r*	*P* value
BMI	0.19	0.127	0.45	< 0.001	0.09	0.487
WHR	0.20	0.100	0.27	0.028	0.04	0.733
BFP	0.20	0.113	0.33	0.007	0.18	0.146
VFC	0.11	0.397	0.30	0.014	0.02	0.904
TG	0.21	0.096	0.22	0.082	−0.16	0.205
TC	0.36	0.003	0.14	0.271	0.17	0.180
HDL	0.13	0.301	0.19	0.135	0.26	0.038
LDL	0.19	0.134	0.02	0.892	0.26	0.036
Adiponectin	0.08	0.515	0.25	0.045	0.28	0.023
Leptin	0.16	0.204	0.17	0.169	0.16	0.202
Visfatin	0.02	0.902	−0.12	0.333	−0.01	0.920
TNF-α	0.08	0.506	0.47	< 0.001	0.24	0.056
IL-6	0.01	0.913	0.32	0.008	0.05	0.696
Homocysteine	0.13	0.300	−0.05	0.675	0.02	0.868
UA	0.07	0.580	0.03	0.827	0.05	0.686

*BFP: body fat percentage; BMI: body mass index; HDL: high-density lipoprotein; LDL: low-density lipoprotein; WHR: waist-hip ratio; TC: total cholesterol; TG: triglyceride; UA: uric acid; VFC: visceral fat content

### FT4

The distribution of body fat content and lipid metabolism according to FT4 are shown in Table 2. We observed significant differences in BMI (*P* = 0.033), WHR (*P* = 0.030), LDL-C (*P* = 0.014), and IL-6 (*P* = 0.009) among the FT4 categories, but there were no significant differences in BFP (*P* = 0.221), VFC (*P* = 0.050), TG (*P* = 0.393), TC (*P* = 0.069), HDL-C (*P* = 0.068), adiponectin (*P* = 0.052), leptin (*P* = 0.129), visfatin (*P* = 0.058), TNF-α (*P* = 0.062), homocysteine (*P* = 0.237), or uric acid (*P* = 0.501). The pairwise comparison showed the following: the lowest category of FT4 was associated with lower BMI, as compared with the middle category of FT4 (MD, − 2.58; 95% CI, − 4.55 to − 0.61; *P* < 0.05); the lower category of FT4 was associated with lower WHR, as compared with the middle category of FT4 (MD, − 0.04; 95% CI, − 0.07 to − 0.01; *P* < 0.05), whereas the middle category of FT4 was associated with increased WHR, as compared with the highest category of FT4 (MD, 0.03; 95% CI, 0.00 to 0.06; *P* < 0.05); the lowest (MD, − 0.79; 95% CI, − 1.34 to − 0.24; *P* < 0.05) and middle categories (MD, − 0.63; 95% CI, − 1.18 to − 0.08; *P* < 0.05) of FT4 were associated with lower LDL-C, as compared with the highest category of FT4; the lowest category of FT4 was associated with lower IL-6, as compared with the middle category of FT4 (MD, − 1.29; 95% CI, − 2.12 to − 0.45; *P* < 0.05), whereas the middle category of FT4 was associated with increased IL-6, as compared with the highest category of FT4 (MD, 0.92; 95% CI, 0.09 to 1.75; *P* < 0.05). Moreover, we noted that FT4 level was positively correlated with HDL-C (*r* = 0.26; *P* = 0.038), LDL-C (*r* = 0.26; *P* = 0.036), and adiponectin (*r* = 0.28; *P* = 0.023) (Table 3).

### TSH

The distribution of body fat content and lipid metabolism according to TSH are shown in Table 2. We noted significant differences in TC (*P* = 0.005) and HDL-C (*P* = 0.010) among the TSH categories, but there were no significant differences in BMI (*P* = 0.163), WHR (*P* = 0.163), BFP (*P* = 0.158), VFC (*P* = 0.613), TG (*P* = 0.291), LDL (*P* = 0.105), adiponectin (*P* = 0.425), leptin (*P* = 0.339), visfatin (*P* = 0.959), TNF-α (*P* = 0.234), IL-6 (*P* = 0.423), homocysteine (*P* = 0.381), or uric acid (*P* = 0.574). The pairwise comparison showed the following: the lowest category of TSH was associated with lower TC, as compared with the middle (MD, − 0.81; 95% CI, − 1.54 to − 0.08; *P* < 0.05) and highest categories (MD, − 1.22; 95% CI, − 1.95 to − 0.49; *P* < 0.05) of TSH; the lowest category of TSH was associated with lower HDL-C, as compared with the middle (MD, − 0.22; 95% CI, − 0.37 to − 0.08; *P* < 0.05) and highest categories (MD, − 0.16; 95% CI, − 0.30 to − 0.02; *P* < 0.05) of TSH. Furthermore, the TSH level was positively correlated with TC (*r* = 0.36; *P* = 0.003).

## Discussion

This study assessed the associations of thyroid hormones with body fat content and lipid metabolism in euthyroid male patients with T2DM. This study found significant differences in BMI, BFP, VFC, adiponectin, TNF-α, and IL-6 among the different FT3 groups; in BMI, WHR, LDL-C, and IL-6 among the different FT4 groups; and in TC and HDL-C among the different TSH groups. Moreover, FT3 was positively related to BMI, WHR, BFP, VFC, adiponectin, TNF-α, and IL-6. FT4 was positively correlated with HDL-C, LDL-C, and adiponectin. TSH was positively correlated with TC.

Studies have reported that thyroid metabolism is closely related to obesity and T2DM even in euthyroid patients [11–14]. However, the complexity of the mechanism is highlighted by different results depending on the populations studied. A previous study from China in patients with diabetes showed that TSH was higher in female than male patients and that TSH was positively associated with serum TC and LDL-C in women with T2DM [15]. In this study, all the subjects were male, and the interference of sex hormones on thyroid function and lipid metabolism could be excluded. Javed et al. observed that hyperthyrotrophin was present in obese adolescents with normal thyroid function [7]. A previous study showed that progressive central fat accumulation was associated with an increase in both FT3 and TSH in women, but this was independent of insulin resistance [16]. This result merits further examination in men. However, the details of the differences in body fat content and lipid metabolism among various thyroid hormones in euthyroid male patients with T2DM was unknown. Therefore, this cross-sectional study was performed to explore any potential role of thyroid hormones on body fat content and lipid metabolism in euthyroid male patients with T2DM.

We observed that FT3 was positively related to BMI, WHR, BFP, VFC, adiponectin, TNF-α, and IL-6 in euthyroid male patients with T2DM. Patients with T2DM presented with long-term absolute or relative insulin deficiency, reduced thyroid iodine uptake, poor thyroid function, and damaged structure [17]. Moreover, thyroxine under the action of type 2 iodothyronine deiodinases could produce FT3, and FT3 could inactivated under the action of type 3 iodothyronine deiodinases. The regulate gene transcription and protein expression through FT3 binds to the thyroid hormone nuclear receptor could affect the development, homeostasis, and regeneration of skeletal muscles [18]. Interesting, we noted the BMI, WHR, and IL-6 was high in the middle FT4 category, while the significant difference mainly observed between middle and lowest FT4 categories. Moreover, our study found that FT4 was positively correlated with HDL-C, LDL-C, and adiponectin. The reason for this could be there was no information on adiposity or muscularity, which could bias the potential correlation between FT4 and LDL-C [19]. Moreover, the association of FT4 and lipid metabolism could mediated by intricate sensing and feedback systems acted at the physiological, metabolic, molecular, and transcriptional levels in liver [20]. Finally, the difference between FT3 and FT4 on body fat content and lipid metabolism could explained by the production process of FT3 and FT4, and the potential role of FT3:FT4 ratio on body fat content and lipid metabolism in euthyroid male patients with T2DM should be evaluated in further large-scale prospective studies.

Importantly, we noted that TSH was positively correlated with TC, but it was not associated with body fat content. Thyroid microstructure disorder exists in patients with T2DM with normal thyroid hormone levels, and the degree of disorder is related to blood glucose level and the insulin resistance index [21]. Adipose tissue is an organ that actively participates in the balance of energy metabolism. Adipose tissue can secrete many adipocytokines to regulate its own functions and that of other tissues, such as adiponectin, leptin, and visfatin. Lu et al. believed that TSH was an important regulator of adipocyte differentiation and TSH may act on adipocytes expressing TSH, thus changing the growth and differentiation of adipocytes and regulating the secretion of various adipokines in adipocytes [22]. However, in this study, TSH was not correlated with body fat content, which may be related to the limitations of the study such as the small number of samples. A larger sample size would allow further quartile analysis.

Several limitations of this study should be acknowledged. First, this study was cross-sectional, and the causality associations of thyroid hormones with body fat content and lipid metabolism could not be established. Second, the severity of T2DM was not addressed, which needed further adjustment of the HbA1c levels. Third, the background treatment strategies for T2DM was not addressed, which might play an important role on body fat content and lipid metabolism. Further prospective study should be performed to verify the causality associations of thyroid hormones with body fat content and lipid metabolism in patients with T2DM.

## Conclusion

This study found that thyroid hormone was positively correlated with body fat content and lipid metabolism in euthyroid male patients with T2DM.

## Data Availability

The datasets used and/or analysed during the current study are available from the corresponding author on reasonable request.

## Abbreviations

T2DM: Type 2 diabetes mellitus
TSH: Thyroid stimulating hormone
FT3: Free triiodothyronine
FT4: Free thyroxine
SCH: Subclinical hypothyroidism
BMI: Body mass index
MBP: Mean blood pressure
HDL-C: High density lipoprotein
LDL-C: Low density lipoprotein
HOMA-IR: Insulin resistance index
